# Characteristics of temperature evolution from 1960 to 2015 in the Three Rivers’ Headstream Region, Qinghai, China

**DOI:** 10.1038/s41598-020-76534-z

**Published:** 2020-11-20

**Authors:** Xiaoqiong Liu, Yuyang Zhang, Yansui Liu, Xinzheng Zhao, Jian Zhang, Yang Rui

**Affiliations:** 1 The Provincial Key Laboratory of Surface System and Environment Capacity, Northwest University, Xi’an, 710127 China; 2 College of Urban and Environmental Sciences, Northwest University, Xi’an, 710127 China; 3 Institute of Geographic Sciences and Natural Resources Research, CAS, Beijing, 100101 China

**Keywords:** Climate change, Climate-change ecology

## Abstract

The cumulative anomaly analysis, the ensemble empirical mode decomposition (EEMD), the Bernaola Galvan heuristic segmentation algorithm (BGSA), the Le Page test, the moving t test at different sub-series scales, and the quasi-periodic oscillations (QPOs) were used to demonstrate the statistical characteristics of the temperature changes in the study area from 1960 to 2015. The results were as follows: the temperatures varied obviously among subregions and seasons and they generally increased; the climate tendency rates of autumn mean temperatures were higher than those of summer and spring; additionally, the temperatures in the three subregions of the Three Rivers’ Headstream Region (THRHR) were relatively low in the 1960s, especially in the early 1960s, followed by those in the 1970s, and the annual mean temperature has been increasing since the mid-late 1980s, especially in the middle 1990s. The results of EEMD showed that the QPOs of the annual mean temperature series in the study area were mainly quasi-3 years, quasi-5–8 years, quasi-12–15 years, and quasi-35–38 years. The results of the annual mean temperature series mutational sites showed that a significant warming mutation began in approximately 1997; and the mutational sites of seasonal mean temperature series in the three subregions of the THRHR all began in the middle and late 1990s. The prediction result of the temperature series trend based on multiple methods showed that the warming persistence of annual and seasonal mean temperature series would be stronger, and their seasonal and regional differences were obvious.

## Introduction

The temporal and spatial variation in temperature in the Qinghai-Tibet Plateau has a significant regional effect, which has a far-reaching impact on the development of glaciers and the evolution of important ecosystems such as marshes and wetlands in this region. In addition, the temperature tendency rate in the Qinghai-Tibetan Plateau is obviously higher, and its mutational time of the temperature series is earlier than that in eastern China^1,2^. The heat source effect of the Qinghai-Tibetan Plateau directly affects climate change in the middle and high latitudes of the Northern Hemisphere^3^, and its temperature changes correlate well with global climate change^4^. Because of the high amplitude of temperature variation and the advancement of climate change^5,6^, climate change in the Qinghai-Tibetan Plateau has always been a hotspot for scholars. Relevant studies show that high-altitude areas are more sensitive to global climate change than low-altitude areas, which further confirms that the Qinghai-Tibetan Plateau is a sensitive, promoter and amplifier region of global climate change^7–9^. Furthermore, the results of the IPCC Fifth Assessment Report show that the mean rising rate of global surface temperature from 1951 to 2012 was 0.12 °C per decade, which is twice as high as the meteorological recordings since 1880; additionally, the land surface temperature (LST), especially the LST in the high latitudes, increases obviously. Moreover, the Third National Assessment Report on Climate Change issues that the mean rising rate of the Chinese LST was 0.21–0.25 °C per decade^10^, which is almost twice that of the global LST, and there is no doubt that the mean rising rate of the Chinese LST is higher than that of the global LST. Therefore, dedicating further attention to climate change in Chinese high-latitude areas, especially in the Qinghai-Tibetan Plateau, is crucial for global climate change.

The THRHR is located in the hinterland of the Qinghai-Tibetan Plateau; it belongs to the north plateau, and its climate changes are different from those in the south plateau. Climate changes in the THRHR, especially temperature changes, will directly influence the area of glaciers and permafrost and water resource security, and then indirectly influence Asian and global climate change; thus, it is crucial to analyse the temperature changes in the three subregions of the THRHR. The relevant studies focusing on temperature changes in the THRHR include climate change trends and mutations^11,12^, spatial and temporal temperature evolution^13^, trends and causes of extreme climate events^14^, distributive patterns of wetlands under climate change^15^, climate change and its driving effect on runoff^16^, ecological environmental problems and environmental effects under climate change^17,18^, the response of precipitation and extreme precipitation to climate change^18^, and the response of vegetation to climate change^19^, etc.

Compared with the existing studies the possible innovations of this paper are as follows: (1) The temperature series are longer, which further enhances the comparability and credibility of the research results while updating relevant research; (2) the use of multiple methods in the order of trend analysis—mutational sites diagnosis—QPOs calculation—trend prediction to enrich the existing research; (3) spatial interpolation analysis based on ANUSPLIN and comprehensive diagnosis of temperature series mutation sites with multi-methods, especially the improved ordered clustering analysis method, has become a highlight of the paper while improving the quality of the paper. Accordingly, this paper seeks to investigate the statistical characteristics of the temperature series in the three subregions of the THRHR from 1960 to 2015 to thoroughly investigate the temperature evolution in the study area and provide references for ecological environmental construction, water resource security, and economic and social sustainable development in the study area, as well as in the middle and lower reaches of the Lancang-Mekong River, the Yellow River and the Yangtze River.

## Study area and data processing

### Study area

**Figure 1:**
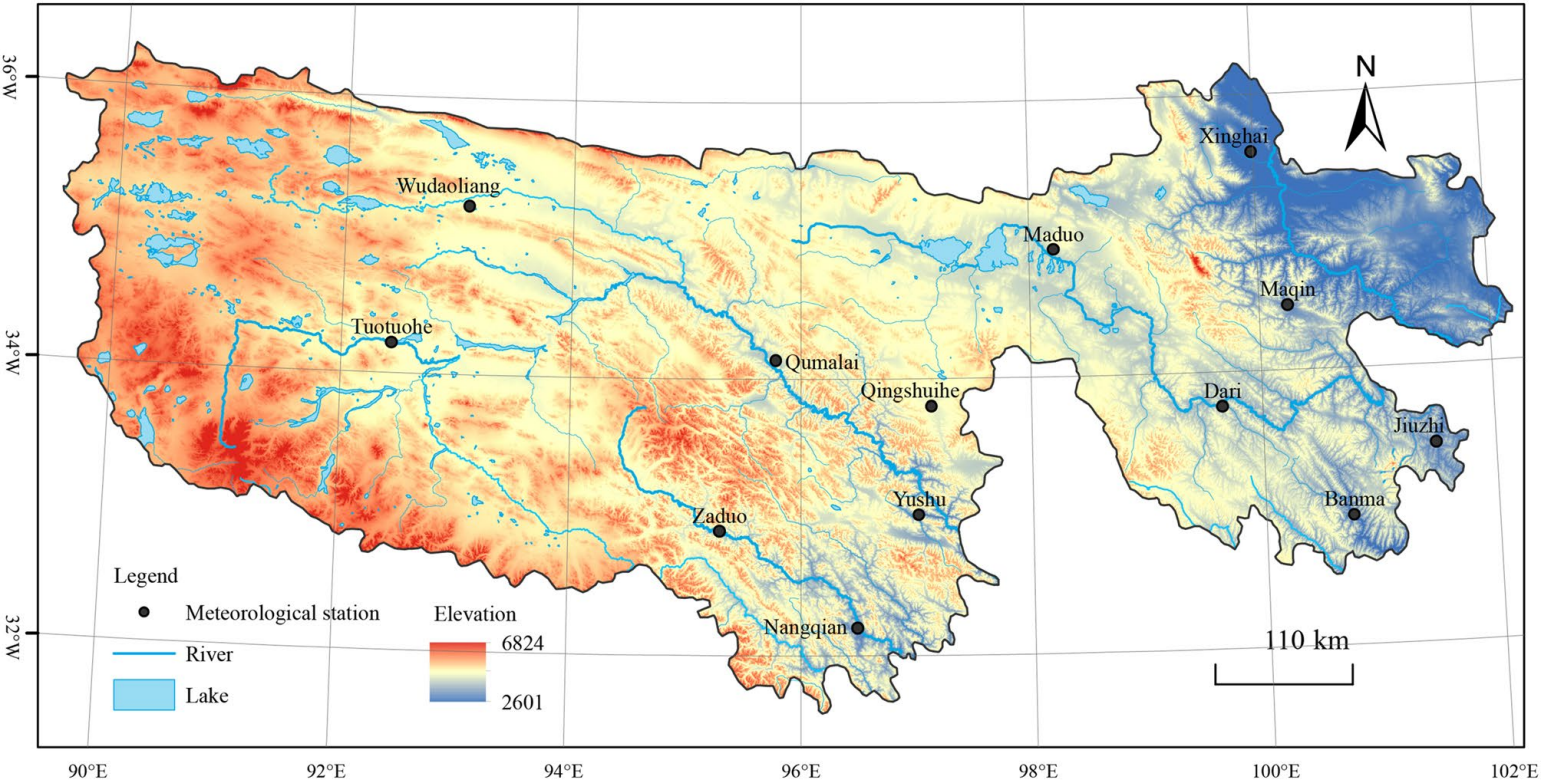
The meteorological stations and their locations in the THRHR ArcGIS 10.5 software^20^ was downloaded from https://support.esri.com/zh-cn/Products/Developers/arcgis-engine/arcgis-engine/10-5-1# overview. It includes the Wudaoliang Town, Tuotuohe Town, Zaduo County, Qumalai County, Nangqian County, Qingshuihe, Yushu County, Maduo County, Xinghai County, Maqin County, Dari County, Jiuzhi County and Banma County.

The THRHR is located in northeast of the Qinghai-Tibetan Plateau (89° 45′–02° 23′ E and 31° 39′−6°12′ N); it is named after the origin of the Lantsang-Mekong River, the Yellow River and the Yangtze River, and its altitude is between 3450 and 6621 m. The study area is vast, and its landforms are complex. Swamp wetlands, glaciers and lakes are widely distributed in it. Moreover, it is an important water resource conservation area and water replenishment area of the Yangtze River, the Yellow River and the Lantsang-Mekong River and an important water source area in China and Asia. Moreover, the ecological barrier to the ecological and environmental security and sustainable development of the middle and lower reaches of the Yellow River and the Yangtze River and its surrounding areas^8^. The study area hosts a typical plateau continental cold climate, where the climate alternates between cold and warm seasons, the dry and wet seasons are distinct, and the daily mean temperature changes greatly while the annual mean temperature varies slightly. According to the regulations of the World Meteorological Organization (WMO) that the mean temperature of the past three decades works as the total annual mean temperature, the annual mean temperature from 1960 to 2015 in the study area is 0.400 6 °C, and the corresponding annual mean precipitation is 500.348 mm. The jurisdiction of the study area includes Guoluo and Yushu Tibetan autonomous prefectures, Xinghai, Tongde autonomous counties in Hainan Tibetan autonomous prefectures, Zeku, He’nan autonomous counties in Huang’nan Tibetan autonomous prefectures, and Tanggula town in Golmud city. The total area of the THRHR is 31.8 × 10^4^ km^2^, accounting for 50.5% of the total area of Qinghai Province (Fig. 1).

### Data sources

There are 18 meteorological stations in total in the THRHR, and considering the different starting times of those meteorological stations and the rescission and removal of some stations, we tried to choose the stations that covered the Lantsang-Mekong River Headstream Region (LARHR), the Yellow River Headstream Region (YERHR) and the Yangtze River Headstream Region (YARHR). Furthermore, we selected the longer temperature series as much as possible. Finally, we chose 13 stations and a total of 56-year span data from January 1st, 1960, to February 29th, 2016. The climate data were derived from the National Meteorological Information Center (https://data.cma.cn/). The missing data interpolation of some meteorological stations was based on the principle of priority of adjacent stations, and they were interpolated by binary linear regression methods. The meteorological stations we analysed in this paper include the Nangqian and Zaduo meteorological stations in the LARHR; the Dari, Xinghai, Maduo, Jiuzhi and Maqin meteorological stations in the YERHR; and the Yushu, Qumalai, Qingshuihe, Wudaoliang, Tuotuohe, and Banma meteorological stations in the YARHR. To analyse the changes in seasonal temperature in the three subregions of the THRHR, the seasons were categorized as follows: spring was from March to May, summer was from June to August, autumn was from September to November, and winter was from December to next February. To reduce seasonal variation in the temperature series and make the data series meet the characteristics of stationary random processes, anomalies were identified and filtered out. In accordance with the relevant regulations of the WMO, the annual mean temperature of 30 years from 1981 to 2010 was selected as the mean annual temperature value used to calculate the annual and seasonal mean temperature anomalies of the study area.

### Methodology

The linear tendency and the moving average method were used to fit the trends of the annual and interannual temperatures in the THRHR. The linear tendency and the moving average method are the simplest and most effective methods for fitting the trend of climate change; the former uses linear tendency to evaluate the climate series correlation, while the latter uses low-pass filtering to determine the climate series correlation^21^. Meanwhile, the multiple linear regression equation was used to fit the relationship between the seasonal tendency rates (their climate tendency rates were relatively higher than those in other seasons) and longitude, latitude and altitude.

1 $$ {\text{y }} = {\text{ a }} + {\text{ bw }} + {\text{ cj }} + {\text{dh}} $$

where y represents the climate tendency rates, w and j represent the longitude and latitude (in decimal), respectively, h represents the altitude (in metres), and a, b, c, d represent regression coefficients.

The promoted ordered cluster analysis, the cumulative anomaly analysis, the Mann–Kendall (M–K) test, the Le Page test and the moving t test based on different scales of subseries were used to diagnose the mutational sites of annual and seasonal mean temperature series in the subregions of the THRHR. Comparatively, the promoted ordered cluster analysis belongs to variance mutation detection; it obeys the principle of keeping the lower deviations in the same cluster while keeping the higher deviations among the different clusters; furthermore, it can identify the edge mutational sites of time series. However, the cumulative anomaly analysis, the M–K test, the moving t test, the Le Page test, and the Bernaola Galvan heuristic segmentation algorithm (BGSA) all belong to the average value mutation detection. Among these, the cumulative anomaly analysis is an intuitive way to judge the trend of a time series by judging the cumulative anomaly and the curve trend. The M–K test is a non-parametric test, in which the samples do not need to obey certain distribution; it can avoid the disturbance of minor outliers, and it is widely used in the mutation detection and the trend analysis of climate change. The Le Page test is a test method for non-parametric double samples that divides the time series into two subseries and treats them as an independent totality in the statistical test, if there is a significant difference between two subseries, it is considered that the datum points that divide the two subseries are the mutational sites. While similar to the principles of the moving t test, this method requires artificially setting the subseries length to avoid the mutational site’s drift caused by the length differences of the subseries, so it is necessary to repeatedly change the subseries length to obtain reliable discrimination^21^. The BG heuristic segmentation algorithm was proposed by Bernaol-Galvan in 2001, and the segmentation idea of this method is to treat the time series as multiple subseries with different mean values and to judge the mutation site by finding out whether the maximum mean difference of each subseries exceeds the different statistical significance^22^. Additionally, the minimum segmentation scale of subseries needs to be ≥ 25a to ensure the validity of the statistical results. Comparatively speaking, there are more than 1–2 mutational sites reckoned by the promoted ordered cluster analysis, while for the cumulative anomaly analysis, there are more than 2–3 sites; the mutational sites reckoned by the M–K test generally contain one, and if there is more than one mutational site, another method is needed to comparatively diagnose. The mutational sites calculated by the moving t test and the Le Page test may vary with the different subseries lengths, and when there is more than one mutational site, it is necessary to integrate other methods to comprehensively diagnose. The BG heuristic segmentation algorithm is one of the most effective ways to detect mutational sites of non-stationary and non-linear series. When there are multiple mutational sites, other similar methods must be integrated for diagnostic needs. In summary, we intend to comparatively diagnose by integrating the above methods to acquire the more accurate mutational sites of the temperature series of the THRHR.

The ensemble empirical mode decomposition (EEMD) is used to determine the QPOs of the temperature series in the THRHR. EEMD is the improved empirical mode decomposition, and here, EMD is the empirical mode decomposition. Compared with traditional Fourier analysis and wavelet analysis, the resolution of the Hilbert spectrum solved by EMD in the time–frequency domain is higher than that of the wavelet spectrum, and its noise-signal ratio is higher than that of traditional Fourier analysis and wavelet analysis. While using EMD analysis, we do not need to specify the primary function of the time series, and it can directly extract the trend component of various scales from the discrete and disordered signal series. The extracted intrinsic mode functions (IMFs) can reflect partial features of the origin signal series in different time scales, and it is suitable to analyse the nonlinear and non-stationary signal series^23^. By using artificial white noise and obtaining the ensemble average value on the basis of EMD^23^, EEMD can avoid the possible mixing phenomenon of signal series caused by EMD, and it has been widely used to detect the frequency domain characteristics of time series^24^ in recent years. In this paper, we extend the data for approximately five years to eliminate the end effect^25^, and 100 groups of white noise disturbances with a noise-signal ratio of 0.02 are added to the normalized temperature series. Then, the smoothing technique is used to filter the singular values that may cause the mixing phenomenon.

In addition, the rescaled range analysis (R/S), the linear tendency, the M–K test and the residual (RES) of EEMD are used to diagnose the persistent intensity of the temperature series in these three subregions. The R/S analysis method is rescaled range analysis, namely, it was proposed by British hydrologist Hurst in analysing the persistent intensity of the hydrological trend of the Nile. It is a non-parametric analysis method used for judging the trend of the climosequence and hydrological series, and its exponent (the H exponent) can reflect the long-term memory process of the time series^26^. When 0 < H < 0.5, it indicates that the series has anti-persistence; when H = 0.5, it indicates that the series conforms to the random walk hypothesis; and when 0.5 < H < 1, it indicates that the series has positive persistence.

## Result analysis

### The spatiotemporal characteristics analysis

#### The temporal characteristics

**Figure 2:**
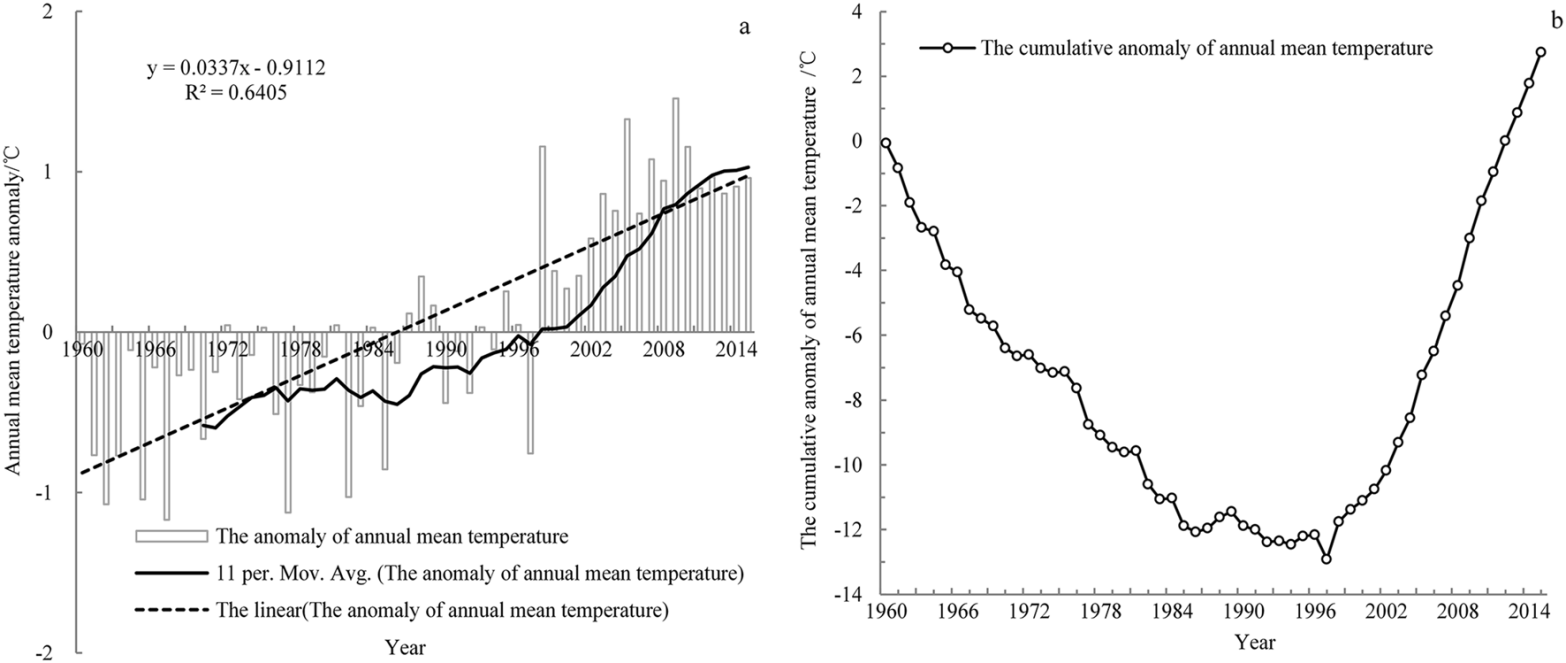
The change in annual mean temperature in the THRHR from 1960 to 2015.

As seen in Fig. 2a, the climate tendency rate of the annual mean temperature series is 0.337 °C per decade, and its correlation coefficient is 0.7674 (> r_0_._01_ = 0.3357). In conjunction with the result of the M–K trend test, this finding demonstrates that the annual mean temperature has significantly increased in the past 56 years. In addition, the climate tendency rates of the annual mean temperature in the LARHR, YERHR and YARHR are 0.352 °C per decade, 0.34 °C per decade and 0.319 °C per decade, respectively (except for the climate tendency rate of the annual temperature in the YARHR, the correlation coefficients of the annual temperature series exceeded the significance level of 0.01). These rates exceed the mean rising rate of annual mean temperature in China (0.21–0.25 °C per decade) and that of the global annual mean temperature (0.07℃ per decade) in the past 56 years. The climate tendency rates of the annual mean temperature in the LARHR and YERHR were higher than those in Northwest China (0.32 °C/10a)^1,27^, and the climate tendency rate of the annual mean temperature in the YARHR was similar to that in Northwest China. The higher annual climate tendency rate of the THRHR is related to the increase in the lowest night temperature in the study area^28^ and the large amount of solar radiation in the lower altitude area of the THRHR^13^; furthermore, it may also be related to the decrease in total cloud cover and the increase in snow cover in the study area^29^. In addition, as seen in Fig. 2a,b, the annual mean temperature of the THRHR has increased significantly since the late1990s.

**Figure 3:**
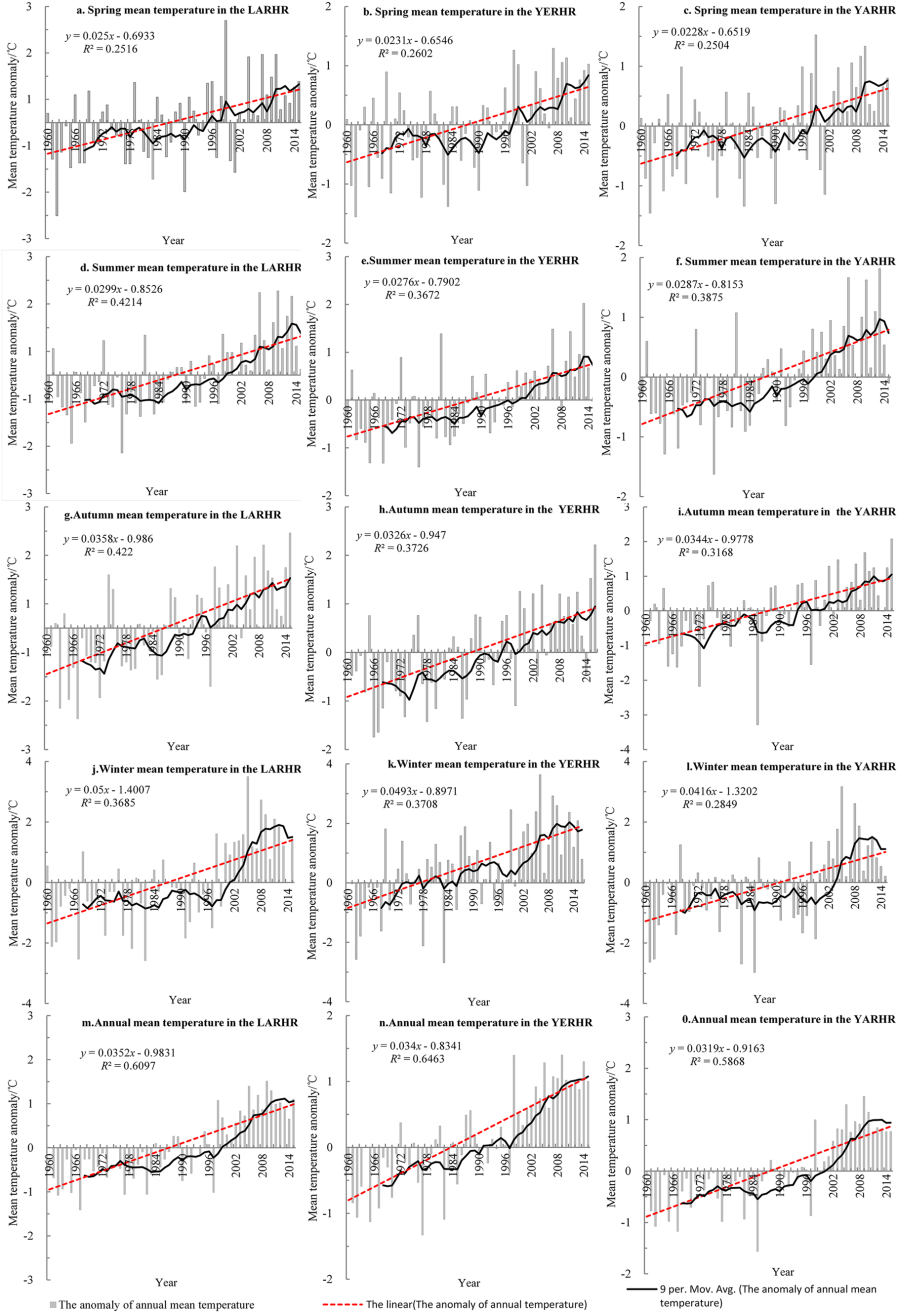
The change in the annual and seasonal mean temperature in the THRHR from 1960 to 2015.

As seen in Fig. 3, the annual and seasonal mean temperatures in the THRHR and its three subregions have been increasing in fluctuations since the 1960s, and the fluctuations in winter and spring are greater than those of summer and autumn. Due to the higher climate tendency rate of the summer, autumn and winter in LARHR than in the other two subregions, the orders of the climate tendency rate of seasonal mean temperature in the THRHR are completely consistent with that of LARHR (autumn, summer, winter, and spring mean temperatures), which are different from that in Northwest China (winter, autumn, spring and summer mean temperatures). In the three subregions, the spring climate tendency rate is lowest, and the orders of the climate tendency rate of autumn and winter are higher than that of spring, while that of summer varies greatly, it is highest in the YARHR, and that of autumn in the LARHR and the YERHR are highest. The high temperature rise rate in autumn and winter in the THRHR is related to the increase in the lowest temperature at night, the climate high tendency rate of winter in three subregions corresponds with the positive phase of the Arctic Oscillation (AO), and namely, the high climate tendency rate of winter corresponds with the high value of the AO. In addition, the annual and seasonal mean temperatures in the LARHR are ≥ 0 °C, respectively; meanwhile, the increasing range of the seasonal mean temperature climate tendency rate is significantly higher than that in the YERHR and YARHR, respectively. The decadal mean temperatures, the decadal minimum and maximum temperatures and their extreme values in the YERHR are lower than those in the LARHR, while they are higher than those in the YARHR (the value of decadal mean temperature is < 0 °C), and the increasing range of the decadal mean temperature in the YERHR is the smallest (Table 1). In comparison, the mean temperature in the YARHR with a higher average altitude is relatively low, while the mean temperature in the LARHR with a lower altitude is relatively high.

**Table 1:** The average, maximum, minimum value and range value of the temperature series in different decades in the Three Rivers’ Headstream Region (°C).

	Area	From 1960 to 2015	1960s	1970s	1980s	1990s	00s in 21st	2010–2015
Mean value	LARHR	2.54	1.95	2.08	2.31	2.49	3.39	3.53
	YERHR	− 0.47	− 1.14	− 0.89	− 0.62	− 0.43	0.27	0.43
	YARHR	− 1.52	− 2.09	− 1.88	− 1.85	− 0.71	− 0.71	− 0.66
Maximum value	LARHR	4.05	2.55	2.59	2.80	3.62	4.05	3.84
	YERHR	0.81	− 0.59	− 0.22	− 0.03	0.81	0.81	0.71
	YARHR	− 0.06	− 1.57	− 1.55	− 1.30	− 0.06	− 0.06	− 0.37
Minimum value	LARHR	1.13	1.13	1.48	1.48	1.53	2.73	3.20
	YERHR	− 1.92	− 1.72	− 1.92	− 1.69	− 0.97	− 0.26	0.29
	YARHR	− 3.08	− 2.69	− 2.50	− 3.08	− 1.33	− 1.33	− 0.75
Range value	LARHR	2.92	1.43	1.11	1.32	2.09	1.33	0.64
	YERHR	2.73	1.14	1.71	1.65	1.78	1.07	0.42
	YARHR	3.02	1.12	0.95	1.78	1.27	1.27	0.38

As seen in Fig. 3, the change in annual mean temperature in the three subregions tends to be consistent with that of the winter mean temperature and summer mean temperature, especially that of the summer mean temperature. The annual and the summer mean temperatures all have been increasing since the mid-1960s. Their increasing rangeability was relatively low in the 1970s–1980s and that of the mid-late 1980s was significant, especially since the mid-1990s. The spring mean temperatures have been increasing since the 1960s; they were relatively low in the 1970s–1980s (the decreasing rangeability of spring mean temperature in the YERHR was the highest), and they have been increasing obviously since the 1980s (especially since the mid-1990s). The autumn mean temperature has been increasing since the 1960s; it changed smoothly in the mid-1970s to 1980s and began significantly increasing in the 1980s (especially since the 1990s). The winter mean temperatures have been increasing since the early 1960s to early 1970s; they decreased from the mid-1970s to late-1980s, and they have been increasing significantly since the 1990s (especially since the mid-1990s). Furthermore, there was a slight cooling trend in approximately 2010. The annual and seasonal mean temperatures during 2010–015 were highest, while their increasing rangeability was smaller than those in the 1990s. Moreover, the occurrence of the weak downward trend of the summer mean temperature in the study area may be related to the local topographic fluctuations at high altitudes, which directly affect the local radiation balance and in turn affect the temperature change^28^.

As seen in Table 2, the climate tendency rates of the annual mean temperature series in the three subregions are all lower than that of the Qinghai Lake basin from 1961 to 2018. Except that the climate tendency rates of the annual mean temperature series of the YERHR are roughly the same, those of the THRHR, the LARHR and the YARHR are higher than those in the north-western region, the Qinghai-Tibet Plateau and the southwestern region. Therefore, although the temperature change rate of the THRHR is lower than that of Qinghai Lake basin due to the various time periods, it is still a relatively warming area compared with the surrounding areas.

**Table 2:** Temperature change rate in the Three Rivers’ Headwaters Region and its surrounding areas.

Region	Time period	Temperature change rate	Region	Time period	Temperature change rate
THRHR	1960–2015	0.337 °C/10a	Qinghai Lake Basin^43^	1961–2018	0.4 °C/10a
LARHR	1960–2015	0.352 °C/10a	Qinghai-Tibet Plateau^28^	1961–2010	0.28 °C/10a
YERHR	1960–2015	0.34 °C/10a	Northwest China^44^	1960–2015	0.32 °C/10a
YARHR	1960–2015	0.319 °C/10a	Southwest China^45^	1960–2016	0.2 °C/10a

### The spatial characteristics

**Figure 4:**
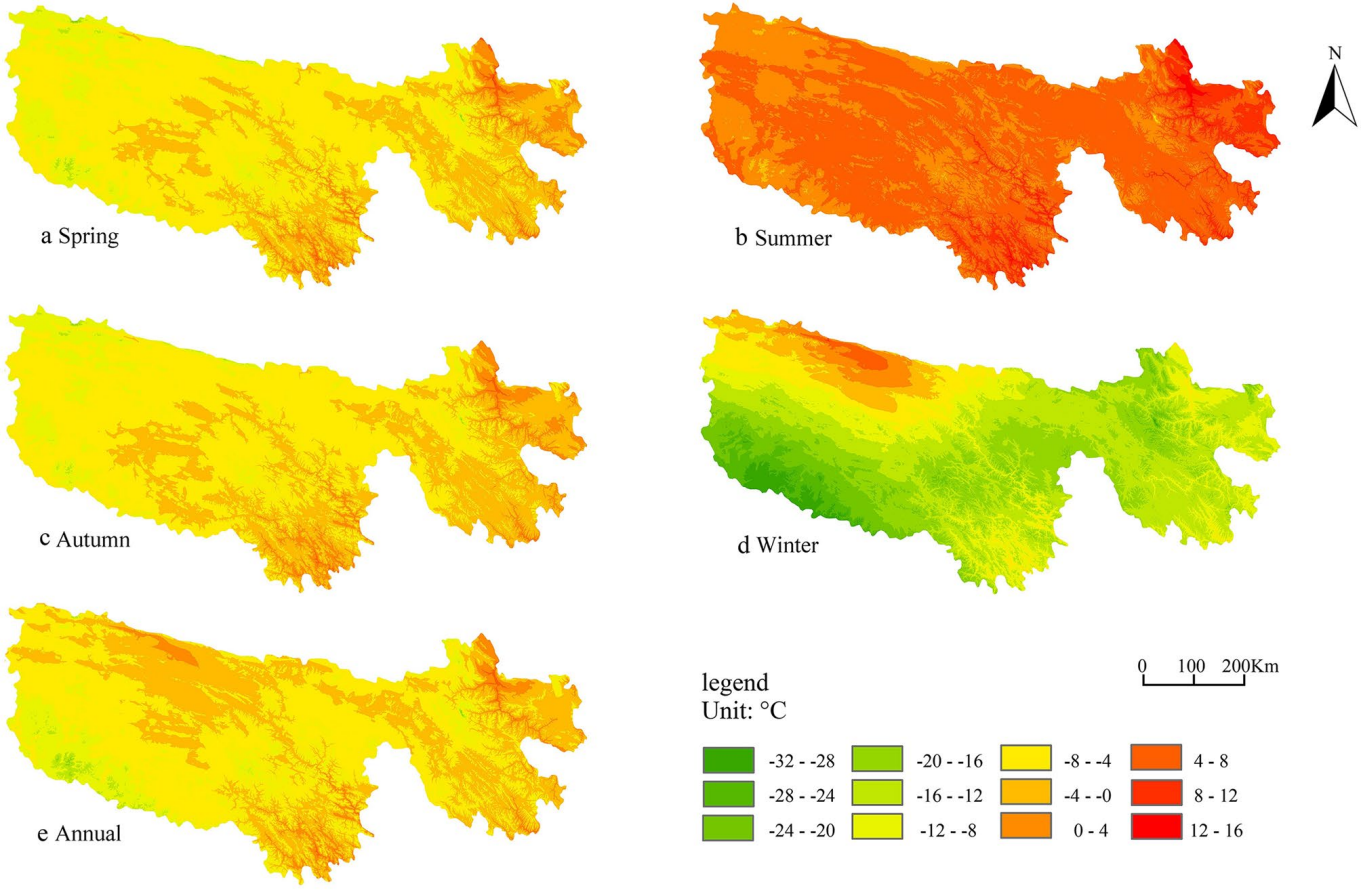
The distribution of seasonal and annual mean temperature in the THRHR from 1960 to 2015 ArcGIS 10.5 software^20^ was downloaded from https://support.esri.com/zh-cn/Products/Developers/arcgis-engine/ arcgis-engine/10–5-1#overview. It includes spring, summer, autumn, winter, and annual mean temperatures in THRHR, and colors can realize the automatic extraction of each data layer.

The spatial distribution of the annual and seasonal mean temperature changes in the THRHR based on ANUSPLIN from 1960 to 2015 is shown in Fig. 4. This interpolation method comprehensively considers the role of concomitant covariates (such as altitude, latitude and longitude, etc.) in temperature change, and it is suitable for the spatial interpolation of meteorology series at high latitudes^30–32^. As seen in Fig. 4, the spatial distribution of the annual mean temperature changes in the THRHR tends to be consistent with that of the winter and autumn mean temperatures, especially the former. (Their climate tendency rates are higher than those of annual, summer and spring mean temperatures).

Affected by the statistical samples and the topography^33^, only the multiple correlation test between the climate tendency rate of autumn mean temperature and altitude exceeds the significance at α = 0.05 among the annual and seasonal mean climate tendency rates from 13 meteorological stations. In other words, the climate tendency rates in the three subregions of the THRHR do not increase consistently with the increase in altitude^34^. As a whole, the climate tendency rates in the three subregions increase with increasing altitude from south to north and from southeast to northwest, and such a situation is much more obvious in the winter. This result aligns with the finding of Tandong Yao et al., who found that the climate warming rangeability in the Qinghai-Tibetan Plateau generally increased with altitude^4^. In terms of the three subregions of the THRHR, except for the climate tendency rate of the winter mean temperature, the annual mean temperature, spring mean temperature, summer mean temperature and autumn mean temperature in the LARHR are higher than those in the YERHR and YARHR, and the spring and autumn climate tendency rates of the YERHR are slightly higher than those in the YARHR; furthermore, their summer climate tendency rates are close, but their winter climate tendency rates are much higher than that in the YARHR.

The multiple linear regression equation is used to fit the relationship between the autumn and winter tendency rates (their climate tendency rates are relatively higher than other seasons) and the longitude, latitude, and altitude. The results show that the confidence test of the two fitted equations exceeds the significance level of 0.05, which again proves that in addition to atmospheric circulation, the topography has a certain impact on the spatial difference in temperature changes in the Qinghai-Tibetan Plateau^35^. The factors affecting the climate tendency rate of autumn include altitude, longitude and latitude (the complex correlation coefficients between the climate tendency rates of autumn mean temperature and altitude and longitude passed the significance test of 0.01, while the multiple correlation coefficients between it and latitude failed the significance test), and those of winter include longitude, latitude and altitude (the complex correlation coefficients between the climate tendency rates of the winter mean temperature and longitude and latitude passed the significance test of 0.01, while the complex correlation coefficient between it and altitude only passed the significance test of 0.1). Furthermore, the correlations of the climate tendency rate of the autumn mean temperature with longitude, latitude and altitude are reversely in line with those of the climate tendency rate of winter, and the further research is needed to study the cause of this opposite trend.

The elevation effect in which the climate tendency rate of the autumn temperature increases with altitude in the study area shows that the melting of ice and snow in high-altitude areas may be affected by the increasing temperature, and the increasing temperature further aggravates the melting of ice and snow^36^. Meanwhile, the surface albedo is effectively reduced, which results in the increasing LST, and then this will further speed up the melting of ice and snow, all of which will lead to the elevation effect on the mean rising rate of the LST. In winter, the surface albedo is high, and the LST is relatively low.

## The mutational analysis

As seen in Table 3, the Spearman test of the annual and seasonal temperature series of the LARHR, YERHR and YARHR are independent; hence, the M–K test can be used to diagnose the mutation sites of the temperature series in the THRHR.

**Table 3:** The Spearman test of the temperature series in the THRHR from 1960 to 2015.

	Annual series	Spring series	Summer series	Autumn series	Winter series
LARHR	0.766	0.523	0.618	0.616	0.565
YERHR	0.809	0.511	0.617	0.605	0.632
YARHR	0.766	0.504	0.643	0.644	0.601

According to the analysis results of the cumulative anomaly analysis, the M–K test, the moving t test, the Le Page test, the promoted ordered cluster analysis and the BGSA, the annual and seasonal mean temperature mutational sites in the three subregions are mainly warming mutational sites (Table 4). In the past 56 years, the mutation time of annual mean temperature in the LARHR, YERHR and YARHR began in 1997a/2004a, 1997a, and 1997a/2002a/1986a, respectively (sorted by the level of mutation strength). The seasonal temperature mutational sites in the three subregions are as follows (the seasonal order is categorized as follows: spring, summer, autumn, and winter): the mutation sites of seasonal temperature in the LARHR began in 1995a, 1997a, 1986a, and 1997a, respectively; the mutation sites of seasonal temperature in the YERHR began in 1995a/2001a, 1996a/1986a, 1986a/1997a, and 1997a/1985a, respectively; and the mutation sites of seasonal temperature in the YARHR began in 1995a/2002a, 1997a/1986a, 1997a/1986a/2000a, and 1999a, respectively. The annual mean temperature mutational sites in the LARHR and the YERHR are basically in line with those of the summer and the winter mean temperature series, and the annual mean temperature mutational sites in the YARHR are basically in line with those of spring and autumn mean temperature series. The strong spring mean temperature mutation sites in the LARHR and YERHR are relatively earlier than those in the YARHR, while the annual and seasonal mean temperature mutational sites in the YARHR are much more complicated than those of the other two subregions. Furthermore, according to the international regulations that state 1755 is the first solar activity cycle, the warming mutations of the annual and seasonal mean temperature series in the three subregions mostly occur around the quiet periods of sunspots.

**Table 4:** The synthetic diagnosis results of the mutation sites of the temperature series in the THRHR from 1960 to 2015.

Methods	The annual mutational sites			LARHR	The seasonal mutational sites			YARHR	
	LARHR	YERHR	YARHR		YERHR				
Cumulative anomaly analysis	1997	1997	1997	**Spring**(1990)	**Summer**(1997)	**Spring**(1995)	**Summer**(1995)	**Spring**(1994)	**Summer**(1995)
				**Autumn**(1986,1997)	**Winter**(1997)	**Autumn**(1997)	**Winter**(1997)	**Autumn**(1987)	**Winter**(1999)
M–K test	1997**	1996***	1997**	**Spring**(1994**,2001**)	**Summer**(1997**)	**Spring**(1997***)	**Summer**(1996***)	**Spring**(1997**, 2002**)	**Summer**(1995**)
				**Autumn**(1993**)	**Winter**(1997**)	**Autumn**(1993**)	**Winter**(1985***)	**Autumn**(1996**)	**Winter**(1999**)
Moving t test	25a and 20a series(-); 15a and10a series (1997***); 5a series (2004*)	25a and 20a series(-); 15a series (1997***); 10a series (1997***); 5a series (1997*, 2004*);	25a,20a and 15a series(-); 10a series (1986**, 1997**, 2002**); 5a series (2002*);	**Spring:** 25a,20a and 5a series(-); 15a series(1995*);10a series **(**1995*);		**Spring:**25a, 20a and 5a series(-); 15a series(1995**);10a series(1990*);		**Spring:**25a,20a,10a and 5a series(-); 15a series(1995**);	
				**Summer:** 25a and 20a series(-); 15a series(1997***);10a series(1986**,1997**); 5a series(1986*);		**Summer:**25a and 20a series(-); 15a series(1997***);10a series(1997**); 5a series(1986*,1997*);		**Summer:**25a and 20a series(-); 15a series(1997***);10a series (1997**); 5a series(1986**,1997*);	
				**Autumn:**25a, 20a and 15a series(-); 10a series(1986**);5a series(1986**);		**Autumn:**25a, 20a and 5a series(-); 15a,10a series(1986**,1997**);		**Autumn:**25a,20a and 5a series (-); 15a series(1986**,1997**); 10a series(1973*,1986*,2000*);	
				**Winter:**25a,20a and 15a series(-); 10a series(1999***);5a series(1987*,1999*)		**Winter:**25a,20a,15a and 5a series(-); 10a series(1999**);		**Winter:**25a,20a and15a series(-); 10a series(1999**);5a series (1999*);	
Le page test	25a and 20a series(-) 15a series (1997**) 10a series (1997*, 2002*) 5a series (2004*)	25a and 20a series(-) 15a series (1997**) 10a series (1997**) 5a series (1997*)	25a,20a and 5a series(-) 15a series (1997**) 10a series (1986*,1997**,2002**)	**Spring**:25a,20a and 10a series(-) 15a series(1994*);5a series (1969*)		**Spring:**25a and 20a series(-) 15a series(1995**);10a series(1995*) 5a series(1995*-1996*,2001*)		**Spring:**25a and 20a series(-);15a series (1995**) 10a series(1994*-1995*,2002*) 5a series(1970*,1995*)	
				**Summer**:25a,20a and 5a series(-) 15a series(1997**);10a series(1997*,2004*)		**Summer:**25a series(-);20a series(1986**) 15a series(1997**);10a series(1997*) 5a series(1997*,2004*)		**Summer:**25a and 5a series(-) 20a series(1986**);15a series(1997**) 10a series(1997**)	
				**Autumn:** 25a,20a,15a and 5a series(-) 10a series(1986**)		**Autumn:**25a and 10a series(-) 20a series(1986*) 15a series (1979*, 1986*, 1993*, 1997**) 5a series(2004*)		**Autumn:**25a and 5a series(-) 20a series (1986**-1987**) 15a series(1993**) 10a series(1973*)	
				**Winter:** 25a,20a and 5a series(-) 15a series(1997**) 10a series(1997**)		**Winter:** 25a series(1985**) 20a series(1985*) 15a series(1997**,1999**) 10a series(1999*) 5a series(-)		**Winter:**25a,20a,15a and 5a series(-) 10a series(1999**)	
BGSA	1998**	1986*,1997*, 1997**	1987*, 1998**	**Spring**(1994**)	**Summer**(1998**)	**Spring**(2002**)	**Summer**(1996**)	**Spring**(1995**)	**Summer**(1996**)
				**Autumn**(1987**)	**Winter**(1970**)	**Autumn**(1987**)	**Winter**(1997**)	**Autumn**(2000**)	**Winter**(2000**)
The promoted ordered cluster analysis	1997***	1997***	1997***	**Spring**(1987***)	**Summer**(1985**)	**Spring**(2001**)	**Summer**(1995**)	**Spring**(1994**)	**Summer**(1986**,1996*)
				**Autumn**(1986**-1987**)	**Winter**(1997**)	**Autumn**(1986**,1993**)	**Winter**(1977**, 1997*)	**Autumn**(1993**,2000*)	**Winter**(1997**)

*Indicates the α = 0.05 significance level.

**Indicates the α = 0.01 significance level.

***Indicates the α = 0.001 significance level.

## The QPO analysis

**Figure 5:**
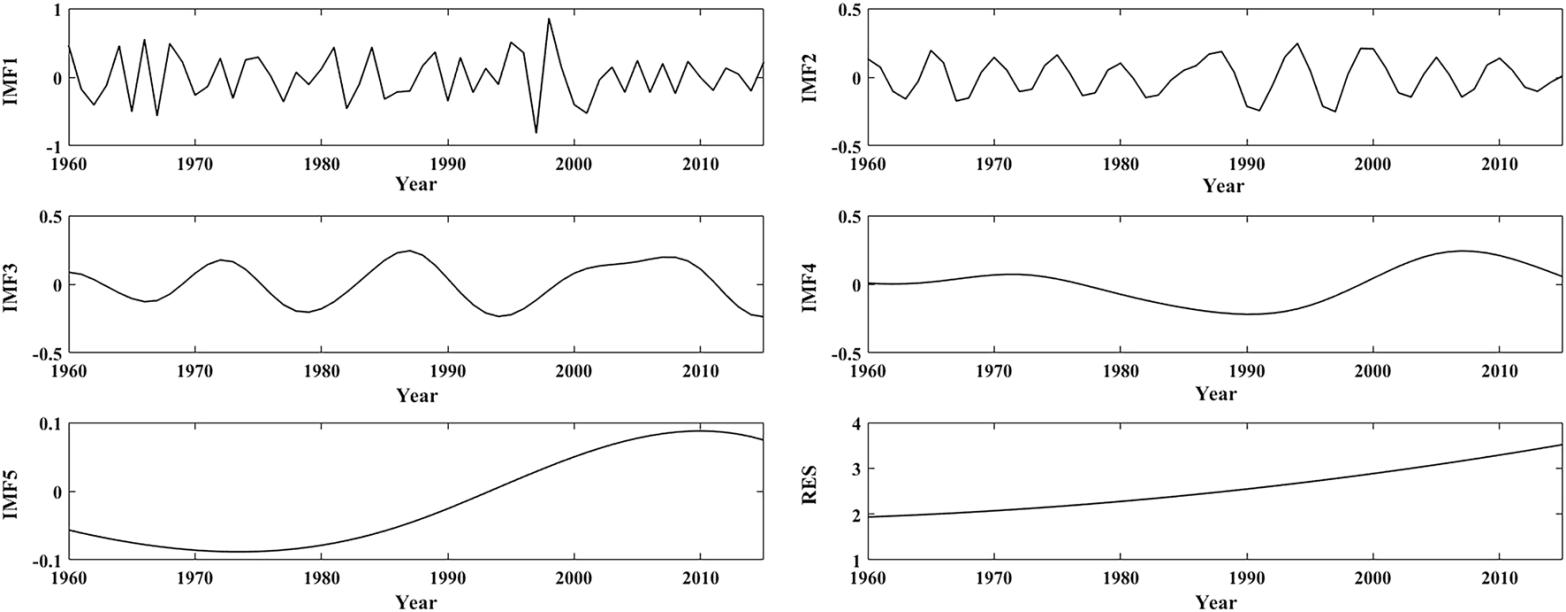
The decomposition of annual mean temperature in the LARHR from 1960 to 2015.

**Figure 6:**
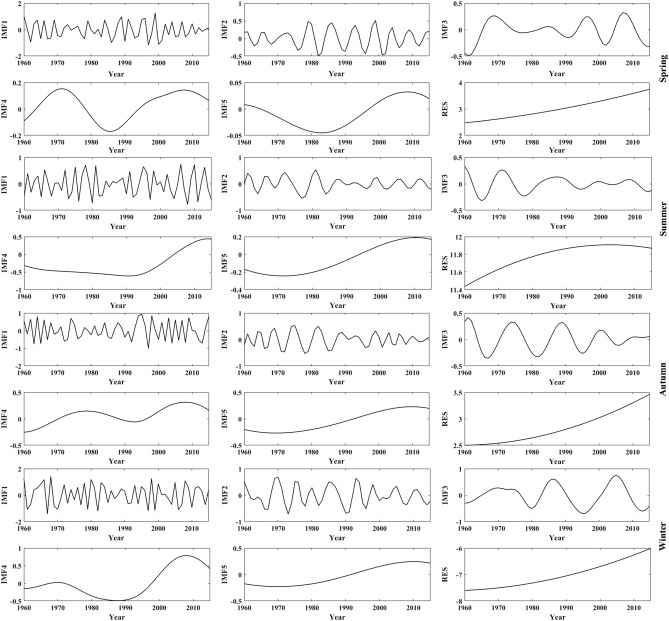
The decomposition of seasonal mean temperature in the LARHR from 1960 to 2015.

**Figure 7:**
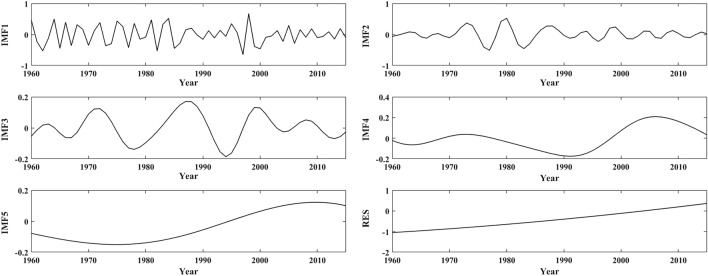
The decomposition of annual mean temperature in the YERHR from 1960 to 2015.

**Figure 8:**
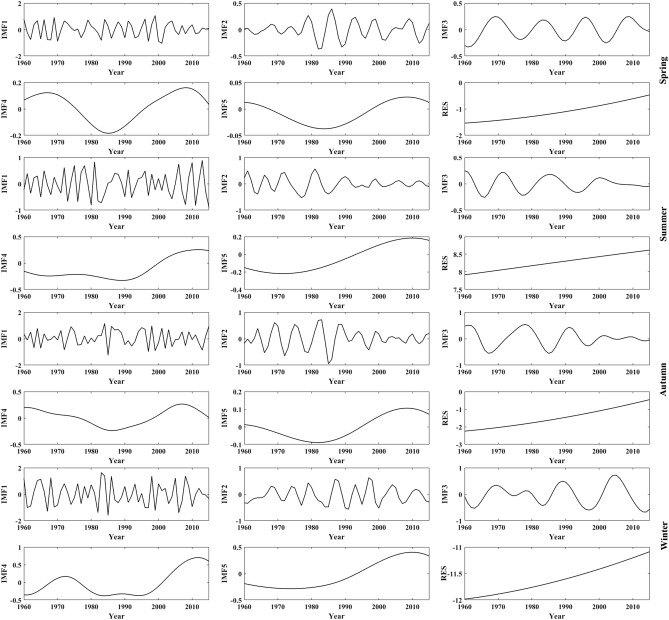
The decomposition of seasonal mean temperature in the YERHR from 1960 to 2015.

**Figure 9:**
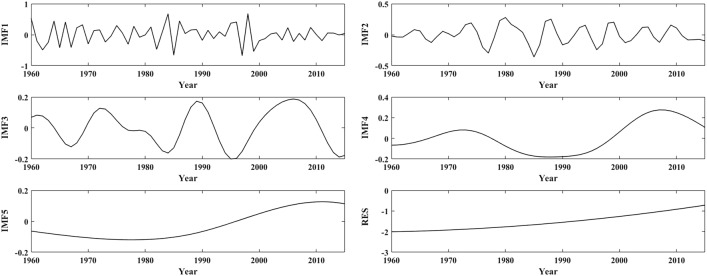
The decomposition of annual mean temperature in the YARHR from 1960 to 2015.

**Figure 10:**
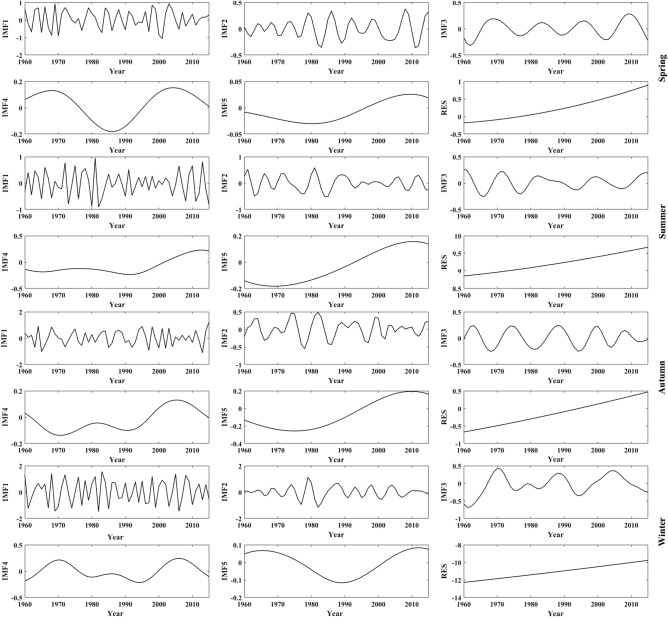
The decomposition of seasonal mean temperature in the YARHR from 1960 to 2015.

Figures 5, 7 and 9 depict the changes in the IMFs and RES of the annual mean temperature anomalies in the three subregions based on EEMD, respectively, and Figs. 6, 8 and 10 depict the seasonal mean temperature decompositions in the LARHR, YERHR and YARHR based on EEMD, respectively. As seen in Table 5, the QPOs, the correlation coefficients and the variance contribution rates of the IMFs of the annual mean temperature are not exactly the same (the variance contribution rates of the IMFs and the RES represent their influences on the original temperature series). Meanwhile, the Spearman test was used to calculate the correlation coefficient of IMFs and the original temperature series and to test its significance.

**Table 5:** The period, correlation coefficient and variance contribution rate of annual mean temperature series in subregions of the THRHR from 1960 to 2015.

	QPO (year)			Correlation coefficient			Variance contribution rate (%)		
	LARHR	YERHR	YARHR	LARHR	YERHR	YARHR	LARHR	YERHR	YARHR
IMF1	3.01	3.26	2.97	0.44***	0.40***	0.42***	28.49	29.16	30.92
IMF2	5.52	7.84	5.26	0.26**	0.31***	0.25**	4.16	11.33	6.30
IMF3	14.55	12.70	11.59	0.27**	0.15	0.27**	5.24	2.24	4.31
IMF4	36.36	34.78	38.1	0.54***	0.55***	0.67***	4.76	3.59	6.71
IMF5	80	88.89	88.89	0.82***	0.78***	0.81***	1.14	3.05	2.71
RES	/	/	/	0.81***	0.81***	0.80***	56.22	50.64	49.06

*Indicates the α = 0.05 significance level.

**Indicates the α = 0.01 significance level.

***Indicates the α = 0.001 significance level.

As seen in Table 5, the QPOs represented by the IMF5 of the annual mean temperature series in the LARHR, the YERHR, and the YARHR are 80a, 89a, and 89a, respectively; except for the obvious difference in amplitude, its phase change is very similar to the RES; hence, such low-frequency IMFs can be combined with the RES to predict the change trend of the time series^37^. In addition, the variance contribution rate of IMF5 of the temperature series of the three subregions is significantly smaller than those of the other four IMFs, which shows that only when the temperature series in the study area is long enough can the QPOs represented by IMF5 be reflected.

As seen in Figs. 7, 8 and Table 5, the annual mean temperature series in the YERHR are mainly determined by the RES, IMF1 and IMF2, and their QPOs are 3.3a, 7.8a, 34.8a, 88.9a and 12.7a, respectively. The main QPOs of the seasonal mean temperature series are 2–4a, 6–a, 11–8a, 28–6a and 50–9a. The spring, autumn and winter mean temperatures are mainly determined by IMF1, RES, and IMF2, and the variance contribution rates of IMF5 in the spring and winter temperature series are relatively low. The summer mean temperature is mainly determined by IMF1, IMF2, and RES, and the variance contribution rates of IMF2 and RES are similar.

As seen in Figs. 9, 10 and Table 5, the change in the annual mean temperature in the YARHR is mainly determined by RES and IMF1, and its main QPOs are 2.97a, 5.26a, 11.59a, 38.1a and 88.89a, respectively. The QPOs of the seasonal mean temperature series are 3a and 6 ~ 7a, and their interdecadal QPOs are 12–17a, 38–2a, and 53–89a. The spring and autumn mean temperatures are mainly determined by RES and IMF1, and the summer mean temperature is mainly determined by IMF1, IMF2 and RES. The winter mean temperature is mainly determined by IMF1, IMF2, and IMF3, and the variance contribution rates of the last two IMFs of the winter mean temperature are similar.

In summary, the change in the annual mean temperature in the study area is mainly determined by the RES and IMF1, while it varies with region and season. The QPOs of the annual and seasonal mean temperature series align with the QPO of the ENSO (3 –7a), while the 11a cycle of sunspot activity has no significant effect on the annual mean temperature. The 80 –89a QPO characterized by the IMF5 indicates that temperature changes in the study area are also affected by solar activity at the quasi-century scale. As seen in Figs. 5, 7 and 9, the trends of the RES show that the changes in annual mean temperature in the three subregions have been increasing since 1960, and the changes in IMF1 in the three subregions show that the interannual change in the annual mean temperature tends to decrease after 2000. Compared with the changes in the annual and seasonal mean temperatures in Fig. 3, the trends of the RES in Figs. 5, 7 and 9 are close to the linear tendency rate of the annual and seasonal mean temperatures.

To verify the climate change response of the three subregions to ENSO events, we chose one article^38^ (Research on the Changes of ENSO events Intensity and Frequency Characteristics in the Past 65 Years) that analysed the occurrence time of the ENSO events from January 1951 to May 2016 to assess the response of the seasonal climate changes in three subregions of the THRHR to El Niño and La Nina events in the past 56 years. The results are as follows:

1. During the El Niño events, the frequency of negative seasonal mean temperature anomalies in the three subregions is slightly higher than that of the positive seasonal mean temperature anomalies (it is most obvious in the YERHR: 21/13), among them cold autumns and cold winters occurred more frequently, and the frequency of negative seasonal mean temperature anomalies of the strongest El Niño year has increased significantly; since the mid-1980s, especially the twenty-first century, the frequency of positive seasonal mean temperature anomalies in the three subregions has increased significantly (especially that of spring, summer and winter); the variations in the seasonal mean temperature anomalies of the LARHR and the YERHR are similar, the frequency of cold winters in these two subregions is higher than that in the YARHR, while the frequency of warm winters is slightly lower; nonetheless, the changes in the autumn mean temperature anomalies in the three subregions are exactly the same.
2. During La Niña events, the frequency of positive seasonal mean temperature anomalies in the three subregions is slightly higher than that of negative seasonal mean temperature anomalies (it is most obvious in the YERHR: 34/24), among them the frequency of warm autumns is the highest, the changes in mean temperature anomalies of spring and summer are roughly the same, and the frequency of positive seasonal mean temperature anomalies of the strongest La Niña events has increased significantly; since the mid-1980s, the frequency of positive seasonal mean temperature anomalies in the three subregions has increased significantly (especially for summer, autumn, and spring); additionally, the variations in the seasonal mean temperature anomalies of the LARHR and the YARHR are similar, and the frequency of cold winters in these two subregions is higher than that in the YARHR.

Overall, except for the similar changes in the mean autumn temperature anomalies, the climate change responses of the three subregions to ENSO events were not the same, yet the negative seasonal mean temperature anomalies in the three subregions occurred more frequently during the El Niño events, when it comes to the La Niña events, it is just the reverse; thus, it can be seen that the ENSO events have a profound impact on climate change in the THRHR. In addition, the interannual oscillation periods of the annual and seasonal mean temperatures of the three subregions are similar to those of ENSO events, which also indicates that ENSO events have a certain impact on the fluctuations of temperature series in the THRHR.

## The predication analysis

The results of the M–K tend test show that the warming trends of the annual and seasonal mean temperatures in the three subregions all greatly exceed the significance level at 0.05, among which the order of the annual and seasonal climate tendency rates in the LARHR is the annual, summer, autumn, winter and spring mean temperatures; the order in the YERHR is the annual, summer, winter, autumn and spring mean temperatures; and that in the YARHR is the annual, autumn, summer, winter and spring mean temperatures. Therefore, the orders of the climate tendency rates of the annual and spring mean temperatures in the three subregions are similar, and the orders of the climate tendency rates of the annual, summer, and spring mean temperatures in the LARHR and YERHR are in line, while those of the autumn and winter mean temperatures are opposite, and the climate tendency rates of the autumn mean temperature in the YARHR are slightly higher than those of the summer mean temperature.

The results of the R/S show that the Hurst exponents of the annual and seasonal mean temperature series in the THRHR are above 0.77, and they are all above level 4 (Table 6)^27^, namely, their climate warming persistence is high, and these values also indicate that the annual and seasonal mean climate warming in the three subregions has strong persistence in the future. In terms of the seasonal mean temperature series, the climate warming persistence of the spring mean temperature is close to that of the annual mean temperature and that of the spring mean temperature is the lowest. The order of the seasonal climate warming persistence in the LARHR is winter, summer, autumn and spring, respectively, and their exponents are close. The order of the seasonal climate warming persistence in the YERHR is summer, autumn, winter and spring, respectively, and the Hurst exponents of the summer and autumn mean temperature series are close, while that of the winter mean temperature is lowest. The order of the seasonal climate warming persistence in the YARHR is summer, winter, autumn and spring, and the Hurst exponents of the winter and autumn mean temperature series are close.

**Table 6:** The Hurst exponent of the annual and seasonal temperature series in the THRHR.

Hurst exponent	Annual mean temperature	Spring mean temperature	Summer mean temperature	Autumn mean temperature	Winter mean temperature
The LARHR	1.00	0.771	0.939	0.916	0.946
The YERHR	0.993	0.782	0.908	0.895	0.856
The YARHR	1.00	0.787	0.954	0.842	0.864

The RES analysis results based on EEMD in the three subregions show that the climate warming in the LARHR is more significant than that in the YERHR and YARHR, and except for the slight climate cooling of the summer mean temperature in the LARHR, the annual and other three seasonal mean temperatures in the three subregions are all rising (especially the winter mean temperature), while the rising trends of the spring, autumn and winter mean temperature are quite different.

Compared with the relevant research that the persistence of climate warming in the Qinghai-Tibetan Plateau is strong based on the R/S analysis^27^, the trend of annual mean temperature in this study is in line with it. In addition, the changes in seasonal mean temperature in this study align with the research done by Jiayang Zhao (the warming trend of the seasonal mean temperature in Northwest China has strong persistence).

## Discussion

The comparison results in this paper show that the temperature changes in the YARHR are in line with those in the Qinghai-Tibetan Plateau^39^. Given the changes in annual mean temperature, the YARHR is a relatively “low-lying land” of global warming, in which its annual mean temperature is relatively low, and its seasonal climate tendency rates are also relatively low. In addition, the warming mutation times in the YARHR are ahead of those in the other two subregions, and the distribution of annual and seasonal temperature mutational sites is more complicated than that in the other two subregions. The annual mean temperature mutational sites in the YARHR are similar to those of the spring and autumn temperature series, while the annual temperature mutational sites in the other two subregions are similar to those of the summer and winter temperature series.

The analysis results that the annual and seasonal mean temperatures in the study area increased significantly over the last 56a are in line with the results of other studies^1,10,13^. This result further confirms that the temperature changes in the study area have been fluctuating but increasing for more than half a century, while there are certain seasonal and spatial differences. The increasing rangeability order of seasonal mean temperature in the study area is mainly the autumn mean temperature followed by the summer and spring mean temperatures. However, if such temperature changes encounter increasing spring and winter precipitation and decreasing autumn and summer precipitation^12,40^, which may affect the cooperation of heat and rain, the net primary productivity of the vegetation may increase in this scenario, and while limited by the specific water conditions, the spring snowstorm incidences may still increase. Moreover, the increasing surface evapotranspiration and vegetation evapotranspiration in the alpine meadow regions may inhibit the productivity of the grassland in the THRHR^41^, and this situation may ultimately have a profound negative impact on the vegetation in the study area.

In comparison, the annual and autumn, winter and spring mean temperatures of the LARHR and the YERHR are higher than those of the YARHR, which may be related to the lower altitude and higher total solar radiation of these two subregions. Meanwhile, the higher annual and seasonal mean temperatures of the LARHR and the YERHR will lead to changes in wetlands and frozen soil in the two subregions ahead of the YARHR and will adversely affect the temperature changes of these two subregions. Moreover, the response of QPOs and their seasonal mean temperature changes to ENSO events in the YERHR are different from those in the LARHR and YARHR, which may be related to the complex topographical conditions in the YEHRH as well as the relatively concentrated human economic activities such as grazing, medicine collection, mining, and urban construction^42^.

Yihui Ding et al. believe that there are some differences in diagnosing the climate mutational sites of the Qinghai-Tibetan Plateau and other parts in China based on the moving t-test, the Yamamoto test, and the M–K test^12^. According to the results of this paper, the cumulative anomaly analysis is the simplest and most intuitive for low-precision analysis; the moving t test is more sensitive than other methods, and its results are relatively close to those of the Le page test. Moreover, the results of the 15a and 10a series and the former are more reliable. Although the analysis principle of the M–K test, especially the BGSA, is relatively complicated, and the M–K test is more suitable for diagnosing the mutational sites of time series where mutations are obvious, the BGSA is more suitable for that of the long-term series, and its results are much more accurate than those of the M–K test. Namely, the mutation analysis results of time series may vary with research methods. In this paper, multiple methods are used to comparatively diagnose the mutational sites of the longer temperature series in the study area. The comparison analysis results show that there are some mutational sites in the annual and seasonal mean temperature series of the three subregions in the past 56 years, which is different from the results reported by Xiangsheng Yi et al. (they thought that there were no mutational sites in the spring mean temperature series^13^). In addition, the analysis results of this paper show that the warming mutation time in the YARHR is earlier than that in the YERHR, which is similar to the research results reported by Jianping Yang^39^, but they think that the warming mutation of the YARHR began in 1971 (the time series of their research started from 1956 to 2000), which is 15 years earlier than that of the warm mutation in our study. Moreover, according to the research in this paper, the strong mutation of annual mean temperature in the YERHR began in 1997a, which is different from the result reported by Yihui Ding et al. (the time series of their research started from 1961 to 2006, and the warming mutation of the annual mean temperature in the Qinghai-Tibetan Plateau began in the mid-1990s). In diagnosing the mutational sites, the results may depend on the number of meteorological stations, the length of the time series and the research methods. Therefore, to effectively improve the accuracy of the analysis results, it is still necessary to integrate multiple methods to carry out a synthetic diagnosis.

The ENSO event has a profound impact on the temperature series changes in the THRHR, and the climate change responses of the three subregions to ENSO events are not the same. In general, from the perspective of the main oscillation periods of the annual mean temperature series in the three subregions, the low-frequency interannual period of ENSO events and the low-value interdecadal period under the influence of sunspot activity may be the main factors that determine the changes in annual mean temperature series in the three subregions.

Furthermore, in the subsequent research, we plan to explore driving factors of the characteristics of spatiotemporal evolution in the temperature in the THRHR, to analyse the effect of synoptic systems, atmospheric circulation and regional human activities on changes of the temperature characteristics in the study area.

## Conclusions

Based on a variety of statistical methods, the conclusions about the temperature evolution in the three subregions of the THRHR in the past 56 years are as follows:

1. The annual and seasonal mean temperatures of the THRHR have demonstrated a fluctuating but clear warming trend, while their regional differences are obvious. The climate tendency rate of the annual mean temperature in the LARHR is slightly higher than that in the YERHR and the YARHR. The regional differences in interdecadal temperature changes across the three subregions are significant, and the annual and seasonal mean temperatures have been increasing significantly since the mid-1980s, especially since the mid-1990s, while the winter and summer mean temperatures have showed a weak decreasing trend since approximately 2010.
2. The correlation test among the winter, autumn mean temperatures (the climate tendency rates of these two seasons are the highest), altitude, latitude and longitude shows that there is a weak negative correlation between the climate tendency rate of the winter mean temperature and altitude, while there is a highly positive correlation between that of the autumn mean temperature and altitude; additionally, the influence of longitude on the climate tendency rate of the winter and autumn mean temperature is greater than that of latitude.
3. The annual mean temperatures in the three subregions of the THRHR are mainly determined by the RES and the low-frequency QPOs under the influence of ENSO events, and the seasonal and regional differences in the QPOs of the seasonal mean temperature are more obvious. The amplitudes of every QPO vary significantly, especially IMF2 and IMF3
4. The mutation sites of the annual and seasonal temperature in the study area are basically warming mutations, the spatial differences in the mutation sites of seasonal temperature are obvious, and the distribution of the annual and seasonal temperature mutational sites in the YARHR are much more complicated than those in the other two subregions. Overall, the annual mean temperature mutations began in approximately 1997, and the seasonal mean temperature mutation roughly began around the middle and late 1990s.
5. The persistent intensity of the trends of the annual and seasonal mean temperature changes based on multiple methods are mainly dominated by strong climate warming, while there are some seasonal and regional differences. The persistent intensity orders of the seasonal temperature variation trend obtained by different methods are slightly different, and among them, the results of the RES analysis are more reliable. Furthermore, the temperature changes in the YARHR are consistent with those of the Qinghai-Tibetan Plateau.
